# Noncontact conductivity and dielectric measurement for high throughput roll-to-roll nanomanufacturing

**DOI:** 10.1038/srep17019

**Published:** 2015-11-23

**Authors:** Nathan D. Orloff, Christian J. Long, Jan Obrzut, Laurent Maillaud, Francesca Mirri, Thomas P. Kole, Robert D. McMichael, Matteo Pasquali, Stephan J. Stranick, J. Alexander Liddle

**Affiliations:** 1 Communication Technology Laboratory, National Institute of Standards and Technology, Boulder, Colorado 80305, USA; 2 Materials Measurement Laboratory, National Institute of Standards and Technology, Gaithersburg, Maryland 20899, USA; 3 Center for Nanoscale Science and Technology, National Institute of Standards and Technology, Gaithersburg, Maryland 20899 USA; 4 Maryland Nanocenter, University of Maryland, College Park, Maryland, 20742, USA; 5 Department of Chemical and Biomolecular Engineering, Department of Chemistry, The Smalley Institute for Nanoscale Science and Technology, Rice University, Houston, Texas 77005, USA; 6 Department of Radiation Oncology, Georgetown University Hospital, Washington, DC 20007, USA

## Abstract

Advances in roll-to-roll processing of graphene and carbon nanotubes have at last led to the continuous production of high-quality coatings and filaments, ushering in a wave of applications for flexible and wearable electronics, woven fabrics, and wires. These applications often require specific electrical properties, and hence precise control over material micro- and nanostructure. While such control can be achieved, in principle, by closed-loop processing methods, there are relatively few noncontact and nondestructive options for quantifying the electrical properties of materials on a moving web at the speed required in modern nanomanufacturing. Here, we demonstrate a noncontact microwave method for measuring the dielectric constant and conductivity (or geometry for samples of known dielectric properties) of materials in a millisecond. Such measurement times are compatible with current and future industrial needs, enabling real-time materials characterization and in-line control of processing variables without disrupting production.

Scalable roll-to-roll and reel-to-reel processing techniques for graphene^1^ and carbon nanotubes^2^ may have a widespread impact on the consumer electronics, nanomanufacturing, and telecommunications industries. Recent applications of nanomaterials, including flexible^3^,^4^ and wearable^5^ electronics, woven fabrics^6^, and wires^2^, herald the eventual industrialization and adoption of nanomaterials. Although several high-throughput structural characterization techniques exist, the nanomaterials community lacks a nondestructive, noncontact electrical characterization technique that does not disrupt production, which may prove critical for these motivating applications. Such tools would enable real-time control of processing variables for quality assurance and characterization, improving the yield and quality of next-generation nanomaterials for devices^7^.

In practice, real-time measurement of roll-to-roll fabricated materials during processing is challenging, because of the speed and vibration of the web^8^. Inspired by work on scanning evanescent microwave microscopy (SEMM)^9^,^10^,^11^,^12^,^13^,^14^,^15^,^16^,^17^, cavity perturbation^18^,^19^,^20^,^21^, and split-ring resonator measurement techniques^22^, we developed a permittivity analysis meter (PAM) that continuously and quantitatively monitors the complex permittivity 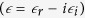 of a material-under-test (MUT) (Supplemental Movie, Fig. 1a). We first verified our system’s performance on a control sample, creating mapping functions between the measured signals to the frequency shift (Δ*f*) and quality factor (Q) relative to the empty cavity. Then, we tested our system at speeds up to 18 cm/s (0.65 km/h) with a model conveyer belt. After measurements on a control sample, we demonstrated our contactless approach on conducting samples by measuring an industrially-relevant double-wall carbon nanotube (DWNT) coating on a polyethylene terephthalate (PET) substrate^23^. We performed all measurements at approximately 10 GHz, the TE_107_ cavity mode.

## Results and Discussion

### Development of the feedback circuit

**Figure 1:**
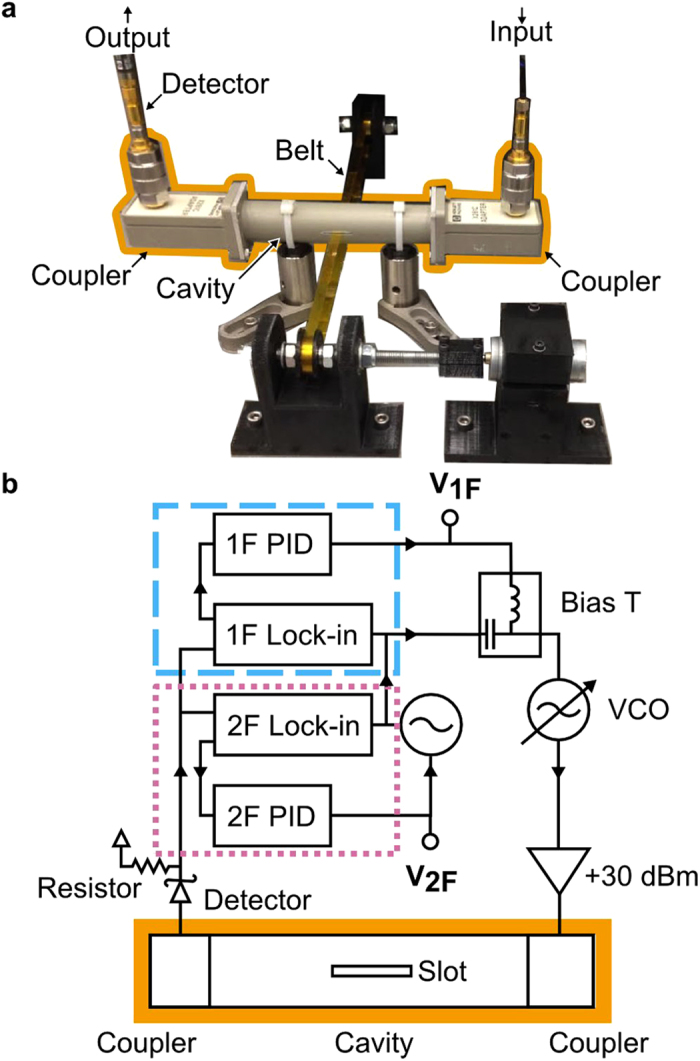
A diagram of the microwave circuit and corresponding photograph of the noncontact conductivity and dielectric constant measurement technique for high-throughput web processing. (**a**) A photograph of the setup. (**b**) The setup uses a voltage-controlled oscillator (VCO) with a frequency (1F feedback loop, blue dashed line) and amplitude (2F feedback loop, pink dotted line) tracking. The cavity is outlined in orange.

The PAM (Fig. 1a) used a feedback circuit (Fig. 1b) to track the changes in the cavity resonance frequency and the Q factor of a cavity in real time. The resonant cavity was excited by a frequency-modulated 10 GHz signal. The modulation frequency was 100 kHz and had a variable modulation amplitude (approximately 1 kHz) around the excitation frequency. The first harmonic of the modulation frequency (100 kHz) was used to lock the center frequency of the excitation to the cavity’s resonance frequency, which was sensitive to the dielectric constant and volume of the MUT. The second harmonic of the modulation frequency (200 kHz) was used to adjust the modulation amplitude so that the range of the output power during a modulation cycle was constant. In this way, the modulation amplitude was related to the Q of the cavity, which was sensitive to the conductivity and volume of the MUT.

Details of the microwave components in the feedback circuit include a voltage-controlled oscillator that was frequency modulated by a lock-in amplifier and connected to a cross-polarized waveguide coupler (Methods). The coupler, in turn, was connected to a 13.5 cm rectangular (X-band) cavity. The cavity contained a slot for the sample cut into the center (Fig. 1b) and a second cross-polarized waveguide coupler that was connected to a microwave diode detector. The diode detector was terminated with a 1 MΩ resistor. Larger terminating resistors improved dynamic range, but also increased the rise time of the diode, in effect trading signal-to-noise for measurement speed. The voltage across the terminating resistor was taken as a measure of the power transmitted through the resonant cavity, and was used as the input for two feedback loops. We then measured two output voltages, V_1F_ and V_2F_, from the feedback loops at 1 ms intervals. In our case, V_1F_ was related to the excitation frequency of the cavity and V_2F_ was related to the modulation amplitude.

### Calibration to reference material

**Figure 2:**
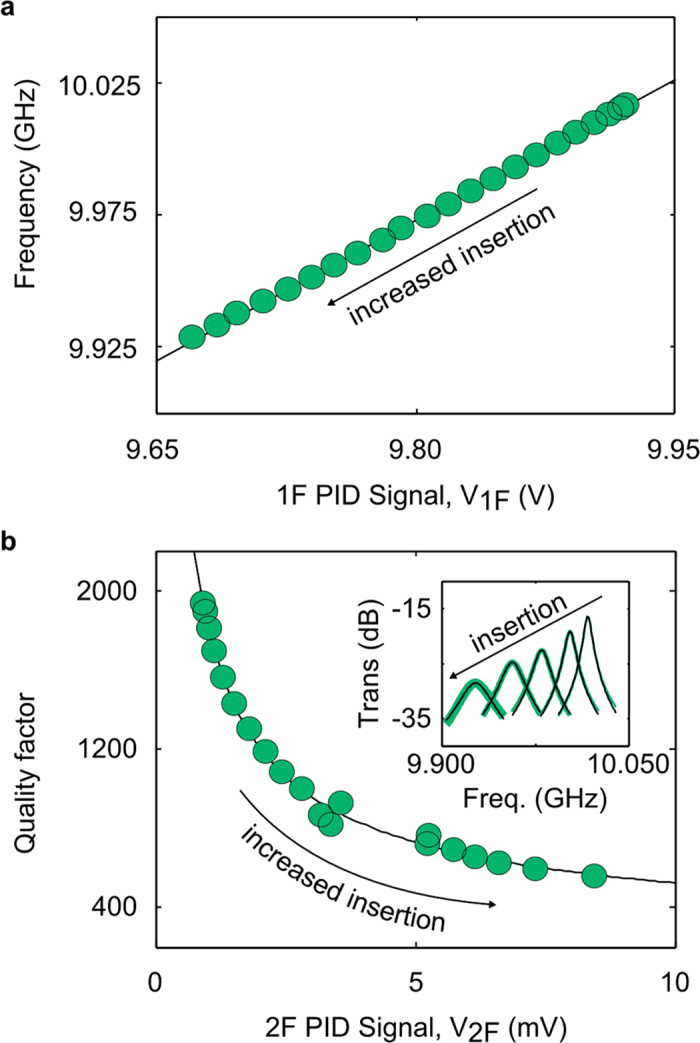
The mapping functions from voltages to frequency and unloaded quality factor. 2 (**a**) The 1F proportional-integrator-derivative (PID) signal (V_1F_) versus the resonant frequency. (**b**) The 2F PID signal (V_2F_) versus the unloaded quality factor for the same samples in (**a**). (**b**) Inset, the magnitude of the transmission of the control sample at variable insertion (green lines) with the harmonic oscillator model (black lines) used to extract the natural frequency and unloaded quality factor. The uncertainties in (**a,b**) were approximately the size of the data circle.

**Figure 3:**
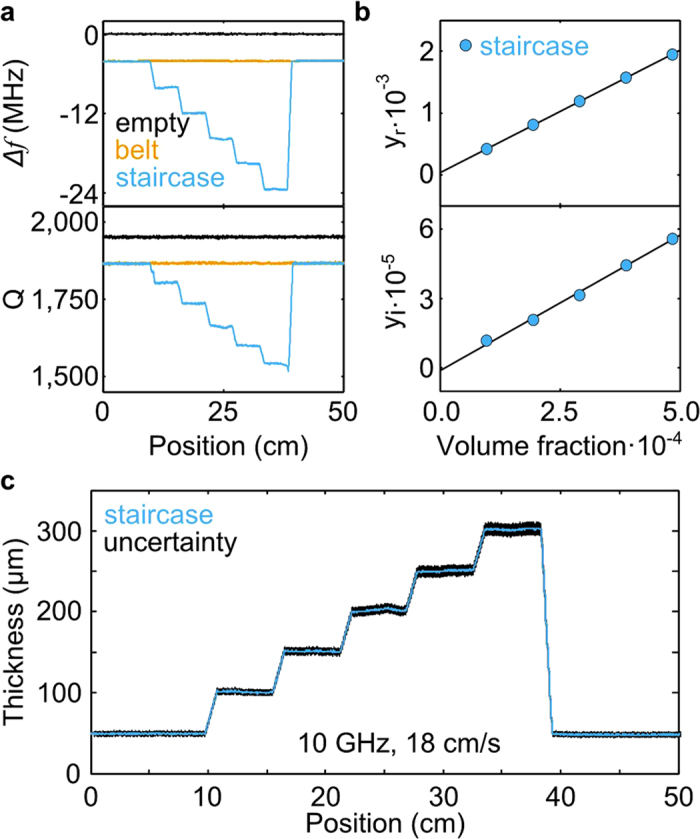
Film thickness and spatial resolution at 18 cm/s of a polyimide sample with a known dielectric constant and conductivity. (**a**) The frequency shift relative to the empty cavity (top) and quality factor (bottom) for the empty cavity (black line), polyimide belt (orange), and polyimide staircase (blue) performed at 10 GHz with a belt speed of 18 cm/s. (**b**) The relative frequency shift (*y*_*r*_) on the top, and the sample damping ratio (*y*_*i*_) on the bottom versus effective sample volume (*x*)^18^. The slopes of the solid black lines are related to the complex permittivity (Methods). (**c**) The sample thickness versus position, using the frequency shift (top, a) and computed dielectric constant (top, b). The thicknesses calculated in (**c**) agreed with measurements made with a caliper. The uncertainty (black) was determined by error propagation of the measured sample dimensions and voltage signal.

We then obtained mapping functions relating V_1F_ and V_2F_ to Δ*f* and Q, respectively (Fig. 2). The choice of sample for this procedure is somewhat arbitrary (Methods), provided that it produces significant changes in Δ*f* and Q. In our case, we used a 3 mm wide nanocomposite sample (*ϵ*_*r*_ = 6.93 ± 0.18, tanδ = *ϵ*_*i*_/*ϵ*_*r*_ = 0.120 ± 0.006), which was characterized following a procedure presented in prior work^18^. We used a translation stage to move the sample to occupy a series of known insertions or fractions of the cavity’s width. The complex transmission parameter (S_21_) was measured with a network analyzer as a function of frequency (inset, Fig. 2b). Next, we fit the S_21_ to a damped harmonic oscillator model^18^. From this fit (black lines in inset, Fig. 2b), we obtained the Δ*f* (x-axis, Fig. 2a) and Q (x-axis, Fig. 2b) as a function of insertion. We repeated the experiment with the high-throughput measurement circuit in place of the network analyzer, generating a mapping function from V_1F_ to Δ*f* (black line, Fig. 2a) and V_2F_ to Q (black line, Fig. 2b). For a 6.4 mm wide and 50 μm thick sample that extended through the center of the cavity, this corresponded to a measurement range of approximately (2 to 50) for the dielectric constant, and approximately (0.02 to 2) S/m for the conductivity. In addition, the contribution of the belt can be corrected for by following the methodology outlines in our previous reports^18^. Once calibrated (Fig. 3), the PAM can measure any unknown sample within the measurement sensitivity and limits of the apparatus.

### Control measurements on dielectrics

To test the PAM at manufacturing-like speeds, we made a control sample from a staircase consisting of one to six levels of 6.4 mm wide by 50 μm thick polyimide strips (Fig. 3). The Δ*f* (top, Fig. 3a) and Q (bottom, Fig. 3a) of the empty cavity, polyimide belt, and staircase were measured at the maximum speed of the model conveyer belt (18 cm/s) and at 10 GHz. The length of each step was nominally 6 cm. We then averaged V_1F_ and V_2F_ over the regions within each step. For each step, we then computed the relative frequency shift (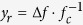), sample damping ratio 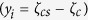, and the effective volume fraction (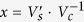) where the 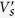 was corrected for the TE_07_ mode shape^18^ (Methods). The volume of the sample 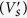 was given as 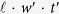, where 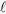 is the depth of the cavity (10.16 mm). The parameters *w*′ and *t*′ are mode-shape corrected width and thickness of the sample^18^. Next, we computed the complex permittivity (Fig. 3b) using the slope technique (Methods)^18^, obtaining a dielectric constant of *ϵ*_*r*_ = (2.980 ± 0.027) with a loss tangent of tanδ = (0.020 ± 0.001) that is in agreement with prior reports^24^. Once we obtained the dielectric constant, we inverted the analytical expression for *y*_*r*_, assumed the reported dielectric constant, and solved for thickness of the sample, as 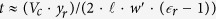, shown in Fig. 3c^24^. Thus, we demonstrated that the Δ*f* and Q (data not shown) can be related to the sample geometry if the dielectric properties are known.

### Verification on conducting thin films

**Figure 4:**
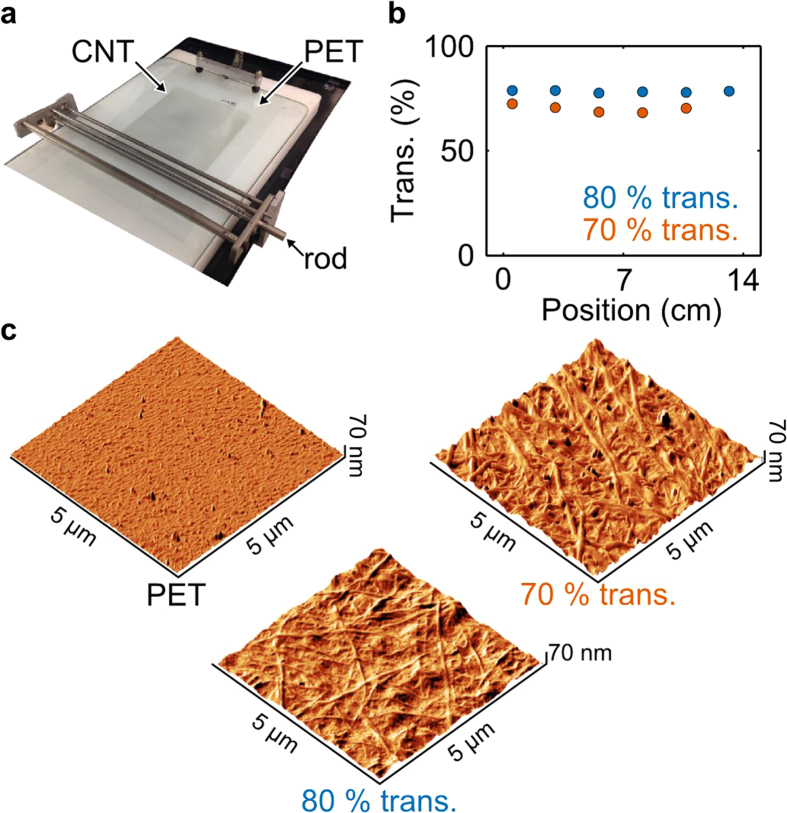
Fabrication of two transparent double-wall carbon nanotube (DWNT) thin films produced by a scalable draw-down Meyer rod coating technique on 120 μm thick polyethylene terephthalate (PET) substrates. (**a**) Photograph of the scalable draw-down rod coating technique. (**b**) Transmittance as a function of position along the sample for the transparent DWNT thin films with nominally 80% and 70% transmittance at a wavelength of 550 nm. The uncertainty in the transmittance was approximately the size of the data circle. (**c**) Atomic force microscopy images of the DWNT thin films on the PET substrate.

After verifying that the PAM can resolve sample geometry changes at industrially relevant processing speeds (more than 0.5 km/h), we tested the PAM’s performance on conducting materials with two DWNT thin films on 120 μm thick PET substrates. We made the films with a scalable draw-down Meyer rod coating technique (Fig. 4a) that is amenable to roll-to-roll processing^23^,^25^. In this approach, the grooved spacing (Methods) on the rod controlled the optical transmittance and sheet resistance. We fabricated two DWNT films with nominal transmittance of 80% and 70% at a wavelength of 550 nm (circles, Fig. 4b). We then confirmed that the nanostructure of the DWNT coatings were uniform and consistent with previous reports by atomic force microscopy (AFM, Methods)^23^. From the AFM images of the topography, we found that both the 80% and 70% transmittance DWNT samples had good uniform coverage.

**Figure 5:**
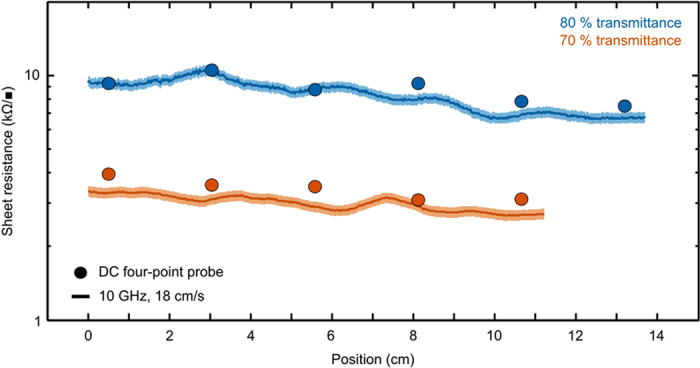
Sheet resistance of two transparent double-wall carbon nanotube (DWNT) thin films produced by a scalable draw-down Meyer rod coating technique on 120 μm thick polyethylene terephthalate (PET) substrates, demonstrating the applicability of the permittivity analysis meter on conducting materials. Data points (circles) are the direct current (DC) sheet resistance measured by four-point probe along the length of the sample. The solid lines are sheet resistance at 10 GHz for a belt speed of 18 cm/s. Uncertainties were computed by error propagation based on uncertainties in the dimensions and voltage signal. The uncertainty in the DC data was approximately the size of the data circle. For the permittivity analysis meter (PAM) data, the shaded region represents the uncertainty.

Finally, we measured both DWNT thin film samples with a direct current (DC) four-point probe (circles, Fig. 5) along the length of the sample to verify our measurement^26^. To measure the samples by the PAM, we cut them into (0.50 ± 0.01) mm wide strips that were approximately 15 cm and 12 cm long for the 80% and 70% films, respectively. We measured the strips by PAM at a speed of 18 cm/s and at frequency of 10 GHz (lines, Fig. 5). We then calculated the sheet resistance as 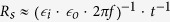, where *ϵ*_*o*_ is the permittivity of free space, *f* is the resonance frequency, and *t* is the total thickness of the sample^18^. Note that for this film and substrate, 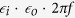 is approximately the conductivity of the sample, because the contribution of the substrate to the microwave power absorption was negligible. For this cavity and a sample with a similar size to the DWNT thin film samples, the range of measureable sheet resistances (Fig. 3b) is approximately (5 to 700) kΩ/■. This explains the larger difference between the PAM and the DC sheet resistance measurement for the 70% film (Fig. 5), which is near the lower-bound limit of detection for this setup. To measure lower sheet resistances, one could optimize the detector (Fig. 1a) or implement an alternative detection scheme to tune the measurement sensitivity. We found that the difference between the sheet resistance of the CNT films measured by the PAM and the DC four-point probe technique was less than twice the uncertainty of the PAM measurement.

## Conclusions

In this paper, we demonstrated a real-time method for measuring the dielectric constant and conductivity at microwave frequencies for samples moving at high speed. We showed that our approach is amenable for manufacturing by demonstrating the technique on a model conveyer belt. We performed a series of control measurements on a polyimide film^24^ with varying thickness, which illustrated the ability to extract the sample geometry. We also applied this measurement technique to industrially relevant carbon nanotube thin films on a polyethylene terephthalate substrate^23^,^27^. Future work will investigate heterodyne detection, in-phase and quadrature mixing, and other approaches to improve sensitivity and decrease measurement time. This method is easily extended to liquids^28^ and gases by integrating a capillary in lieu of a belt. To accommodate wider samples or webs, ongoing work includes Fabry-Perot and dielectric resonators, eliminating the need for the sample to pass through the cavity. Summarizing, we have devised a real-time, quantitative, nondestructive method for dielectric constant and conductivity monitoring of materials in roll-to-roll processing without disrupting production.

## Methods

### Cavity Perturbation

Recently, it was shown that the cavity perturbation equation^29^,^30^ for a sample in an electric field maximum of a cross-coupled cavity could be rewritten as,


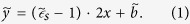


In (1)^18^, the perturbation (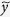) on the resonance in the cavity is complex. The real part of the perturbation (relative frequency shift) can be approximated as the difference between the resonance frequency of the cavity (*ω*_*c*_) and the resonance frequency of the cavity with the sample (*ω*_*cs*_) divided by the resonance frequency of the cavity. The imaginary part of the perturbation (sample damping ratio) can be approximated as the difference between the damping ratio of the cavity (*ζ*_*c*_) and the damping ratio of the cavity with the sample (*ζ*_*cs*_). After making these approximations, we can express the perturbation as,


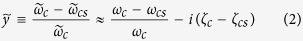


where the damping ratio is related to the quality factor by 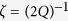. Nonuniform fringing fields contribute to (1) as a small complex intercept (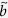). This occurs when a sample does not completely fill the cavity cross-section and when a cavity has a slot in the sidewalls to insert the sample (Fig. 1a). Finally, the effectively volume fraction (*x*) is the ratio of the effective volume of the sample (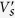) to the volume of the cavity (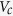),


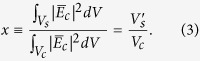


The mode shape in the cavity modifies the effective volume of the sample (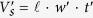), where 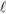 is the length of the sample, *w*′ is the effective width of the sample, and *t*′ is the effective thickness of the sample^18^,^31^.

Previously, we made several important observations from (1)^18^. Among them, the perturbation is linearly proportional to the effective volume fraction, leading to the slope technique. Following the slope technique, a linear regression of the perturbation versus the effective volume fraction (Fig. 3b) yields two slopes that can be related the complex permittivity by (1). Thus, the perturbation (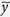) sets the measurement limits, rather than the sample’s complex permittivity or size alone. A detailed uncertainty budget is provided elsewhere^18^; it has been established that the dominant source of uncertainty in this technique is the sample dimensions.

### Feedback Circuit

The first feedback loop tracked the cavity resonance frequency by nulling the modulation of the cavity output power at the modulation frequency, as shown previously^15^. This loop included a lock-in amplifier (1F lock-in) referenced to the modulation frequency of the VCO. The in-phase output of the 1F lock-in was used as the error input to a PID loop (1F PID), and the analog output of the 1F PID (V_1F_ ) was routed through the low frequency arm of a Bias T connected to the VCO. The second feedback loop locked the frequency modulation amplitude to a fraction of the cavity bandwidth by maintaining a constant power modulation amplitude at the second harmonic of the modulation frequency. This loop included a lock-in amplifier (2F lock-in) referenced to the second harmonic of the modulation frequency of the VCO. The quadrature output of the 2F lock-in was used as an input to a PID loop (2F PID) with a constant set-point. The output of the 2F PID loop (V_2F_) was used to control the amplitude of the 100 kHz modulation signal, which was then sent through the high-frequency arm of the Bias T to the VCO. Finally, the PID loop outputs V_1F_ and V_2F_ were measured at 1 ms intervals with a data acquisition board (DAQ).

### Carbon Nanotube Inks

We used stock 0.1% by mass fraction DWNTs that were purchased from a commercial vendor. We dispersed and stabilized the DWNTs in water using 1% by volume a non-ionic commercial surfactant. We homogenized the dispersion by tip sonication for one hour at 22 W (acoustic power). Then, we added the commercial buffer to the aqueous dispersion to reach 3% by mass fraction of DWNTs, which was stirred for 15 minutes.

### Coating Method and Characterization

The rod coating apparatus is a commercial automated draw-down machine, consisting of a glass drawdown pad and several stainless steel coating rods with wires of different diameter wound around them (Meyer rods #3, 6, and 9)^32^. We made the films by dispensing ~1 mL of the CNT ink on a 120 μm thick PET substrate, previously cleaned with isopropanol. Next, we spread the CNT ink onto the substrate by the rod translating over the substrate at 3 cm/s. We dried the CNT film in an oven at 90 °C for 30 min, after which we rinsed the films in a water/ethanol bath (1:1 by volume) for about 30 min. We then oven-dried the CNT films again at 90 °C for 30 min. We measured the *R*_*s*_ with a DC four-point probe and the transmittance with a UV-VIS spectrometer at a wavelength of 550 nm.

### Atomic Force Microscopy

Atomic force microscopy (AFM) and the cantilever were purchased from commercial vendors. Images were acquired using tapping mode in ambient conditions. The cantilever had a spring constant of approximately 30 N/m and a resonant frequency of approximately 300 kHz. The free oscillation amplitude of the tip was approximately 100 nm, and the imaging set-point ratio was 90%. The linear tip speed during scanning was approximately 25 μm/s.

## Additional Information

**How to cite this article**: Orloff, N. D. *et al.* Noncontact conductivity and dielectric measurement for high throughput roll-to-roll nanomanufacturing. *Sci. Rep.* **5**, 17019; doi: 10.1038/srep17019 (2015).

## Supplementary Material

Supplementary Movie

## References

[b1] Bae S. *et al.* Roll-to-roll production of 30-inch graphene films for transparent electrodes. Nat. Nanotechnol. 5, 574–578 (2010). 20562870 10.1038/nnano.2010.132

[b2] Behabtu N. *et al.* Strong, Light, Multifunctional Fibers of Carbon Nanotubes with Ultrahigh Conductivity. Science 339, 182–186 (2013). 23307737 10.1126/science.1228061

[b3] Cao Q. *et al.* Medium-scale carbon nanotube thin-film integrated circuits on flexible plastic substrates. Nature 454, 495–500 (2008). 18650920 10.1038/nature07110

[b4] Eda G., Fanchini G. & Chhowalla M. Large-area ultrathin films of reduced graphene oxide as a transparent and flexible electronic material. Nat. Nanotechnol. 3, 270–274 (2008). 18654522 10.1038/nnano.2008.83

[b5] Shim B. S., Chen W., Doty C., Xu C. & Kotov N. A. Smart Electronic Yarns and Wearable Fabrics for Human Biomonitoring made by Carbon Nanotube Coating with Polyelectrolytes. Nano Lett. 8, 4151–4157 (2008). 19367926 10.1021/nl801495p

[b6] Yamada T. *et al.* A stretchable carbon nanotube strain sensor for human-motion detection. Nat. Nanotechnol. 6, 296–301 (2011). 21441912 10.1038/nnano.2011.36

[b7] Morse J. Nanofabrication Technologies for Roll-to-Roll Processing. (2012) Date of access: 09/09/2014.

[b8] Pagilla P. R., Siraskar N. B. & Dwivedula R. V. Decentralized Control of Web Processing Lines. IEEE Trans. Control Syst. Technol. 15, 106–117 (2007).

[b9] Soohoo R. F. A Microwave Magnetic Microscope. J. Appl. Phys. 33, 1276–1277 (1962).

[b10] Stranick S. J. & Weiss P. S. A tunable microwave frequency alternating current scanning tunneling microscope. Rev. Sci. Instrum. 65, 918–921 (1994).

[b11] Wei T., Xiang X.-D., Wallace-Freedman W. G. & Schultz P. G. Scanning tip microwave near‐field microscope. Appl. Phys. Lett. 68, 3506–3508 (1996).

[b12] Gao C., Wei T., Duewer F., Lu Y. & Xiang X.-D. High spatial resolution quantitative microwave impedance microscopy by a scanning tip microwave near-field microscope. Appl. Phys. Lett. 71, 1872 (1997).

[b13] Takeuchi I. *et al.* Low temperature scanning-tip microwave near-field microscopy of YBa2Cu3O7−x films. Appl. Phys. Lett. 71, 2026–2028 (1997).

[b14] Gao C. & Xiang X. D. Quantitative microwave near-field microscopy of dielectric properties. Rev. Sci. Instrum. 69, 3846–3851 (1998).

[b15] Steinhauer D. E. *et al.* Quantitative imaging of sheet resistance with a scanning near-field microwave microscope. Appl. Phys. Lett. 72, 861–863 (1998).

[b16] Steinhauer D. E. *et al.* Quantitative imaging of dielectric permittivity and tunability with a near-field scanning microwave microscope. Rev. Sci. Instrum. 71, 2751–2758 (2000).

[b17] Imtiaz A., Pollak M., Anlage S. M., Barry J. D. & Melngailis J. Near-field microwave microscopy on nanometer length scales. J. Appl. Phys. 97, 044302 (2005).

[b18] Orloff N. D. *et al.* Dielectric Characterization by Microwave Cavity Perturbation Corrected for Nonuniform Fields. IEEE Trans. Microw. Theory Tech. 62, 2149–2159 (2014).

[b19] Krupka J. & Strupinski W. Measurements of the sheet resistance and conductivity of thin epitaxial graphene and SiC films. Appl. Phys. Lett. 96, 082101 (2010).

[b20] Hao L. *et al.* Non-contact method for measurement of the microwave conductivity of graphene. Appl. Phys. Lett. 103, 123103 (2013).

[b21] Shaforost O. *et al.* Contact-free sheet resistance determination of large area graphene layers by an open dielectric loaded microwave cavity. J. Appl. Phys. 117, 024501 (2015).

[b22] Rowe D. J., Porch A., Barrow D. & Allender C. J. Novel Coupling Structure for the Resonant Coaxial Probe. IEEE Trans. Microw. Theory Tech. 60, 1699–1708 (2012).

[b23] Dan B., Irvin G. C. & Pasquali M. Continuous and Scalable Fabrication of Transparent Conducting Carbon Nanotube Films. ACS Nano 3, 835–843 (2009). 19354279 10.1021/nn8008307

[b24] Oliver G. Using Flex in High-Speed Applications. The PCB Magazine 4, 90–96 (2014).

[b25] Hu L., Kim H. S., Lee J.-Y., Peumans P. & Cui Y. Scalable Coating and Properties of Transparent, Flexible, Silver Nanowire Electrodes. ACS Nano 4, 2955–2963 (2010). 20426409 10.1021/nn1005232

[b26] Smits F. M. Measurement of Sheet Resistivities with the Four-Point Probe. Bell Syst. Tech. J. 37, 711–718 (1958).

[b27] Mirri F. *et al.* High-Performance Carbon Nanotube Transparent Conductive Films by Scalable Dip Coating. ACS Nano 6, 9737–9744 (2012). 23038980 10.1021/nn303201g

[b28] Rubinger C. P. L. & Costa L. C. Building a resonant cavity for the measurement of microwave dielectric permittivity of high loss materials. Microw. Opt. Technol. Lett. 49, 1687–1690 (2007).

[b29] Bethe H. & Schwinger J. in Perturbation Theory for Cavities. NDRC Report D1-117. (Cornell University, 1943).

[b30] Waldron R. A. Perturbation theory of resonant cavities. Proc. IEE - Part C Monogr. 107, 272–274 (1960).

[b31] Higginbottom D., Howes M. J. & Richardson J. R. An Experimental Technique to Evaluate the Complex Permittivity of Small Samples of Microwave Substrates. in 16th European Microwave Conference, 1986. 790–795 (1986), doi: 10.1109/EUMA.1986.334289

[b32] MacLeod D.M. in Coatings Technology: Fundamentals, Testing, and Processing Techniques 3^rd^ edn (ed. Tracton A. A. ) Ch. 19, 144–151 (CRC Press, 2006).

